# Using Age-Stage Two-Sex Life Tables to Assess the Suitability of Three Solanaceous Host Plants for the Invasive Cotton Mealybug *Phenacoccus solenopsis* Tinsley

**DOI:** 10.3390/plants13101381

**Published:** 2024-05-16

**Authors:** Khaled Abbes, Ahlem Harbi, Emilio Guerrieri, Brahim Chermiti

**Affiliations:** 1 Department of Biological Sciences and Plant Protection, High Agronomic Institute of Chott-Mariem, University of Sousse, Sousse 4042, Tunisia; abbes.kaled@live.fr (K.A.); harbi.ahlem@hotmail.fr (A.H.); 2 Institute for Sustainable Plant Protection, National Research Council of Italy, URT IPSP-DISIT, 15121 Alessandria, Italy; emilio.guerrieri@cnr.it

**Keywords:** tomato, potato, eggplant, polyphagous pest

## Abstract

*Phenacoccus solenopsis* Tinsley (Hemiptera: Coccomorpha: Pseudococcidae), the cotton mealybug, is an invasive polyphagous species that has been extending its geographic range, posing a conspicuous threat to many Mediterranean crops of economic importance. These include three species of Solanaceae, namely *Solanum lycopersicum* L. (tomato), *Solanum tuberosum* L. (potato) and *Solanum melongena* L. (eggplant) all of which are economically important worldwide. In this study, we used age-stage two-sex life tables to investigate the suitability of these three plant species as hosts for *P. solenopsis* and to calculate pest fitness, life history parameters and population projection parameters. All tested host plants that were suitable for the pest and eggplant host plant induced a higher fecundity (276.50 ± 10.78 eggs/female), net reproductive rate (*R*_0_) (243.32 ± 15.83 offspring/female) and finite rate of increase (*λ*) (1.18 ± 0.0043 day^−1^) and more extended adult longevity (males: 6.50 ± 0.34 days and females: 24.15 ± 0.50 days). Population growth predictions over a period of 90 days of infestation, commencing with an initial population of 10 eggs showed that adult population size was 674,551 on tomato, 826,717 on potato and 355,139 on eggplant. Our data on plant host preference of *P. solenopsis* will aid the development of appropriate management strategies and achieve successful control of this invasive pest in key Mediterranean crop systems.

## 1. Introduction

Invasive insect pests remain a constant threat to agricultural production systems worldwide. Their invasion benefits from both climate change and the rapid global trade of agricultural products including ornamentals, fruits and seedlings. In most cases, their establishment outside their native range can cause significant economic and ecological losses by disturbing ecosystem balance and impairing already implemented Integrated Pest Management (IPM) strategies, often leading to the overuse of synthetic insecticides [1,2,3].

Among invasive agricultural pests, mealybugs (Hemiptera: Coccomorpha: Pseudococcidae) are a major threat due to their biological features including cryptic behavior, high reproductive capacity, ecological plasticity and, in many cases, the ability to develop resistance to insecticides [4,5]. The cotton mealybug *Phenacoccus solenopsis* Tinsley has expanded its geographical distribution over the last decades [6]. Thus far, it has been reported in more than 70 countries worldwide and it is considered one of the most devastating pests of cotton in Asia (e.g., China, India, Iran and Pakistan) [7,8,9]. More recently, this pest has invaded several Mediterranean countries including Algeria, CyprusEgypt, France, Greece, Israel, Italy, Morocco, Tunisia and Turkey [10,11,12,13,14,15,16,17].

*Phaenacoccus solenopsis* is a highly polyphagous sap-feeding insect attacking about 300 host plant species belonging to 65 families, in particular species of Amaranthaceae, Asteraceae, Cucurbitaceae, Euphorbiaceae, Fabaceae, Lamiaceae, Malvaceae and Solanaceae. It primarily feeds on the aerial parts of the plants, but can attack also roots and collars, producing abundant wax and honeydew, the latter promoting the development of sooty mould. Mealybug infestations cause distorted and bushy shoots, crinkled and/or twisted and bunchy leaves and ultimately death in the absence of efficient control measures [8,9,14,18].

Currently, cotton remains the preferred host crop of *P. solenopsis* with yield losses in Pakistan, China and India of 0.48, 1.4 and 1.12 million tons, respectively, during 2008–2009 [18]. Hence, an intensive and irrational use of insecticides for its management was adopted, leading to the emergence of resistance to many insecticides in several field populations [19,20,21,22]. In other invaded regions where cotton is not a key crop, *P. solenopsis* spread adapting to wild and ornamental hosts such as *Lantana camara* L. (Verbenaceae) and *Hibiscus rosa-sinensis* L. (Malvaceae) or has become a serious pest of Solanaceous crops, as has happened in Israel and Egypt where it has been reported on bell pepper, eggplant, tomato and potato [11,23,24]. Owing to the recent invasion of *P. solenopsis* in European and Mediterranean countries, it is therefore critical to assess the suitability of most relevant Solanaceous vegetables as host plants to reduce the possible yield losses and to develop efficient monitoring and control protocols.

Life table analyses including life history parameters and population fluctuations are an essential tool for studying insect ecology and fitness. They provide helpful information about insect biology and reproductive capacity allowing prediction of their performance and mortality patterns in relation to different host plants and environmental conditions [25]. However, for biparental species, conventional age-specific life tables often omit the contribution of males and differentiation by stage [26]. To fill this gap, Chi and Liu [27], Chi [28] and Chi et al. [29] developed the age-stage two-sex life table which has been successfully applied to study various ecological aspects of insect pests and their natural enemies [30,31,32].

In this study, we investigated the biology, survival, reproduction and life table parameters of *P. solenopsis* when reared on three economically important Solanaceous host plants, *S. lycopersicum* (tomato), *S. tuberosum* (potato) and *S. melongena* (eggplant), based on the age-stage two-sex life table using the TWOSEX-MSChart^®^ software. We also assessed population-projection parameters using the TIMING-MSChart^®^ program.

## 2. Results

Values for the developmental time of each stage, longevity and total duration of the life cycle of males and females on different hosts are presented in Table 1.

The egg-incubation time was the same on all host plants (1 d). For females, the duration of the first instar was longer on eggplant (6.18 ± 0.12 days) than on potato (5.58 ± 0.11 days) and tomato (4.88 ± 0.13 days) (*P_TP_* = 0.0001; *P_TE_* = 0; *P_PE_* = 0.0005). The duration of the second instar did not differ among host plants ranging from 4.70 ± 0.15 to 4.84 ± 0.18 days (*P_TP_* = 0.701; *P_TE_* = 0.8721; *P_PE_*= 0.1412). The duration of the third instar was greater on eggplant (7.09 ± 0.24 days) than on tomato (6.30 ± 0.26 days) and potato (6.28 ± 0.20 days) (*P_TP_* = 0.0202; *P_TE_* = 0.0287; *P_PE_* = 0.0111). For males, the duration of the first instar was greater on eggplant (6.50 ± 0.43 days) than on potato (5.36 ± 0.15 days) and tomato (4.71 ± 0.36 days) (*P_TP_* = 0.1045; *P_TE_* = 0.0035; *P_PE_* = 0.0185). The duration of the second instar was greater on potato (6.54 ± 0.41 days) than on tomato (5.28 ± 0.47 days) and eggplant (5.16 ± 0.48 days) (*P_TP_* = 0.047; *P_TE_* = 0.8721; *P_PE_* = 0.1412). The duration of the pupa was greater on potato (7.09 ± 0.54 days) and tomato (6.71 ± 0.47 days) than on eggplant (4.50 ± 0.22 days) (*P_TP_* = 0.602; *P_TE_*= 0.0001; *P_PE_*= 0.0001). The total female pre-adult developmental duration was greater on eggplant than tomato and potato (*P_TP_* = 0.0717; *P_TE_* = 0; *P_PE_* = 0.001) and that of male was greater on potato than on tomato and eggplant (*P_TP_* = 0.0082; *P_TE_* = 0.5937; *P_PE_* = 0). The longevity of adults was greater on eggplant than on tomato and potato for both males (*P_TP_* = 0.9248; *P_TE_* = 0; *P_PE_* = 0) and females (*P_TP_* = 0.0008; *P_TE_* = 0; *P_PE_* = 0). The duration of the life cycle of females was greatest on eggplant and lowest on tomato and potato (*P_TP_* = 0.3548; *P_TE_* = 0; *P_PE_* = 0) and that of males was the longest on potato and eggplant and the shortest on tomato (*P_TP_* = 0.0168; *P_TE_* = 0.0203; *P_PE_* = 0.5512).

Data on fecundity, adult preoviposition period (APOP), total preoviposition period (TPOP) and oviposition days on the different host plants tested are shown in Table 2.

The greatest APOP was registered on eggplant followed by tomato and potato (*P_TP_* = 0.0001; *P_TE_* = 0; *P_PE_* = 0). Similarly, TPOP was greater on eggplant than on tomato and potato (*P_TP_* = 0.4267; *P_TE_* = 0; *P_PE_* = 0). Concerning fecundity, it was greater on eggplant (276.50 ± 10.78 eggs/female) than on potato (244.92 ± 9.75 eggs/female) and on tomato (155.60 ± 11.99 eggs/female) (*P_TP_* = 0; *P_TE_* = 0; *P_PE_* = 0.0298). The number of oviposition days was greatest on potato (13.64 ± 0.17 days) followed by tomato (12.72 ± 0.24 days) and eggplant (10.84 ± 0.11 days) (*P_TP_* = 0.0022; *P_TE_* = 0; *P_PE_* = 0).

The mean net reproductive rate (*R*_0_) of *P. solenopsis* was significantly different on the selected host plants, attaining its highest value on eggplant (243.32 ± 15.83 nymphs/female), followed by potato (191.04 ± 16.23 nymphs/female) and tomato (133.82 ± 12.78 nymphs/female) (*P_TP_* = 0.0056; *P_TE_* = 0; *P_PE_* = 0.0213). The average intrinsic rate of increase (*r*) was greatest on potato (0.18 ± 0.00 d^−1^) compared to tomato and eggplant (*P_TP_* = 0.0245; *P_TE_* = 0.5411; *P_PE_* = 0.0024). The finite rate of increase (*λ*) was significantly greater on tomato and eggplant (1.18 ± 0.005 d^−1^ and 1.18 ± 0.004 d^−1^, respectively) than on potato (1.20 ± 0.004 d^−1^) (*P_TP_* = 0.0244; *P_TE_* = 0.5411; *P_PE_* = 0.0024) (Table 3).

**Figure 1:**
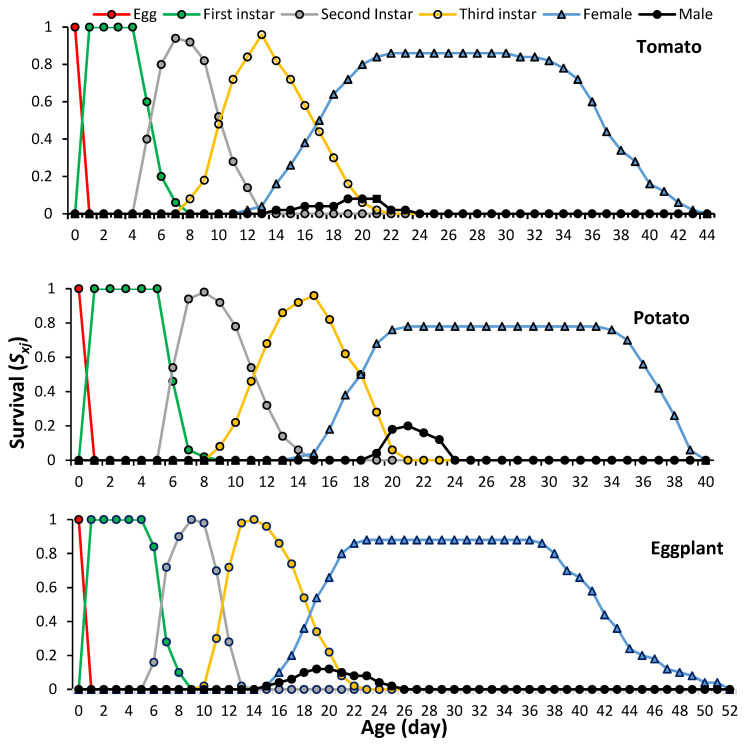
Survival rate of different development stages of *Phenacoccus solenopsis* on different host plants (25 ± 1 °C, 60 ± 5% RH and 16:8 h (L:D)).

The age-stage-specific survival rate (*s_xj_*) of *P. solenopsis* on different host plants indicates the probability that a newborn will survive to age *x* and develop to stage *j* (Figure 1).

Due to variable developmental rates among individuals, significant overlap was observed between stages in the survival curves. The first females emerged on days 12, 14 and 15, while the first males appeared on days 14, 19 and 16 on tomato, potato and eggplant, respectively. The survival rates of preadult instars ranged between 92% and 100%, while those of females were 84%, 78% and 86% on tomato, potato and eggplant, respectively. The lowest survival rate was recorded for males not exceeding 20% on all studied host plants.

**Figure 2:**
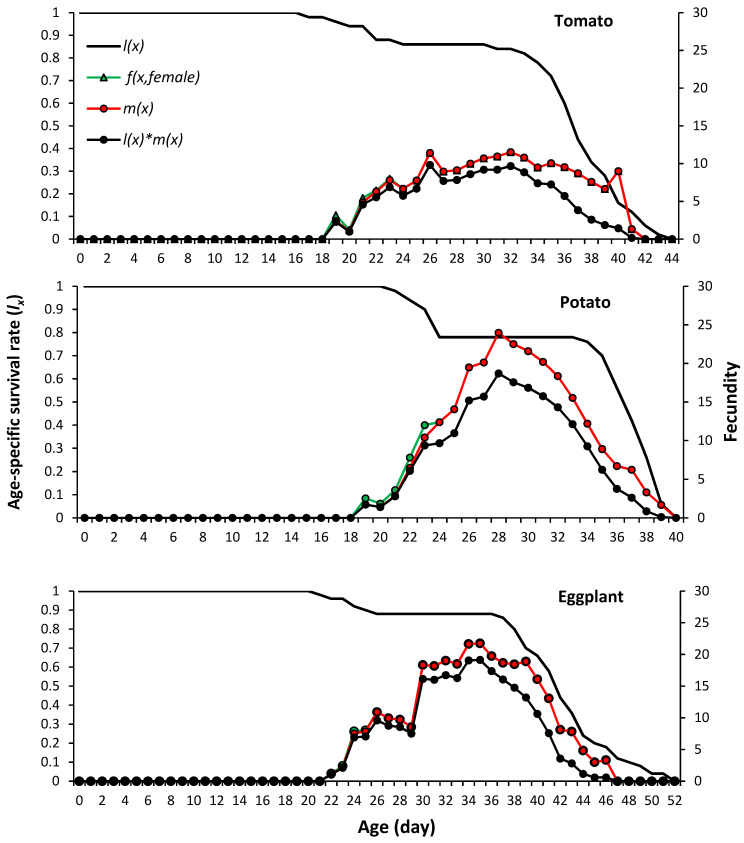
Age-specific survival rate (*l_x_*), female age-specific fecundity (*f_x_*), age-specific fecundity (*m_x_*) and age-specific maternity (l_x_*m_x_) versus age of *Phenacoccus solenopsis* on different host plants (25 ± 1 °C, 60 ± 5% RH and 16:8 h (L:D)).

The single age-stage survival rate (*l_x_*) predicts that an egg will survive to age *x* (Figure 2). On all host plants, the *l_x_* curve was constantly around 100% during the early stages, indicating a relatively low mortality rate.

The age-stage-specific fecundity (*f_x_*) curve peak on potato was greater than that recorded for eggplant and tomato. The curve of age-specific fecundity (*m_x_*) showed that reproduction began at 19 days on tomato and potato and 3 days later on eggplant, and that the fecundity on potato and eggplant was greater than on tomato.

**Figure 3:**
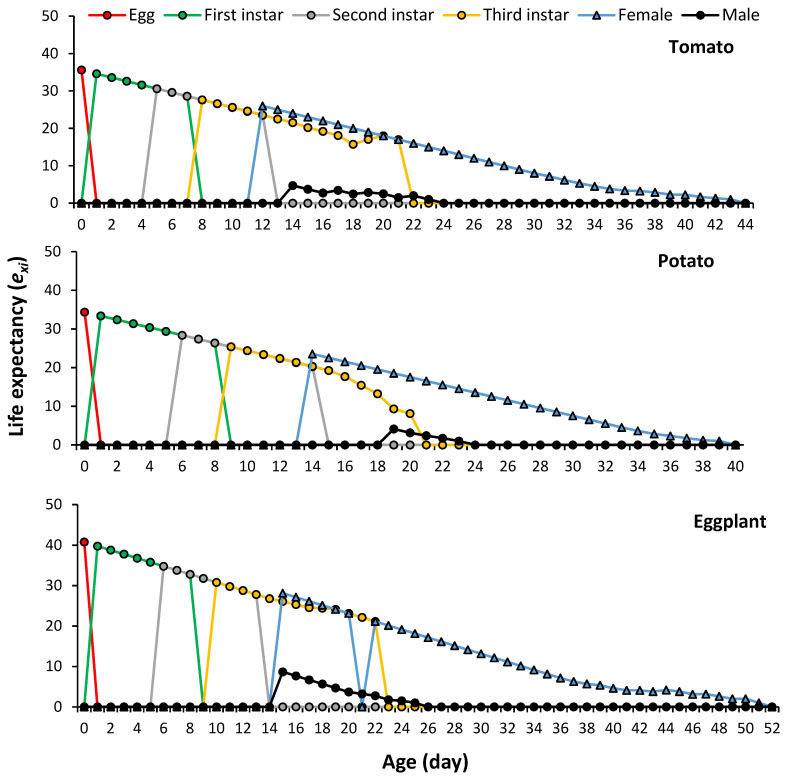
Age-stage life expectancy of *Phenacoccus solenopsis* on different host plants (25 ± 1 °C, 60 ± 5% RH and 16:8 h (L:D)).

The age-stage life expectancy (*e_xj_*) estimates the life duration of an individual of age *x* and stage *j*. The longevity of *P. solenopsis* at age zero (*e*_01_) was 35.6 days on tomato, 34.38 days on potato and 40.80 days on eggplant (Figure 3).

**Figure 4:**
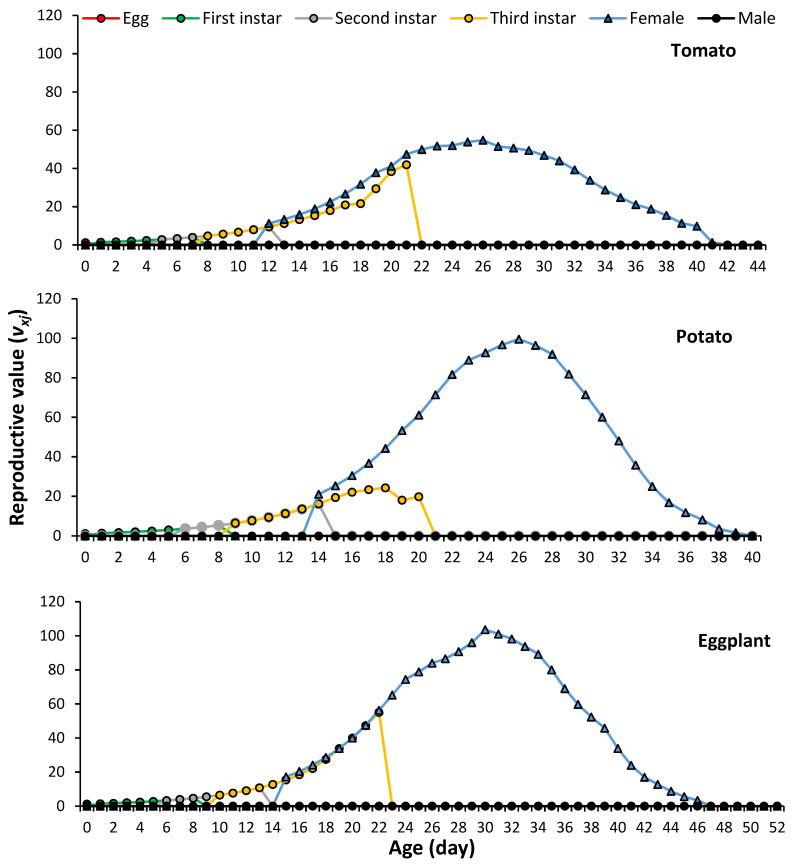
Age-stage reproductive value of *Phenacoccus solenopsis* on different host plants (25 ± 1 °C, 60 ± 5% RH and 16:8 h (L:D)).

The age-stage reproductive value (*v_xj_*) shows the contribution of an individual from age *x* to stage *j* to the future population. The curves of reproductive value significantly increased when reproduction began, as shown in Figure 4. The value of *v_xj_* peaked on day 26 for tomato and potato and on day 30 for eggplant with values of 54.87 d^−1^, 99.57 d^−1^ and 103.59 d^−1^, respectively.

**Figure 5:**
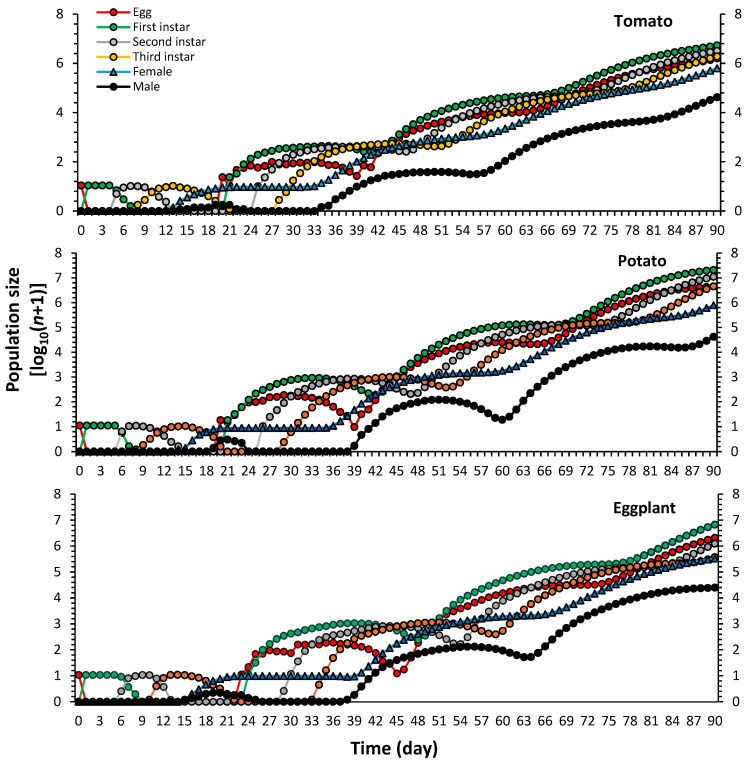
Population growth predictions of *Phenacoccus solenopsis* on different host plants.

**Figure 6:**
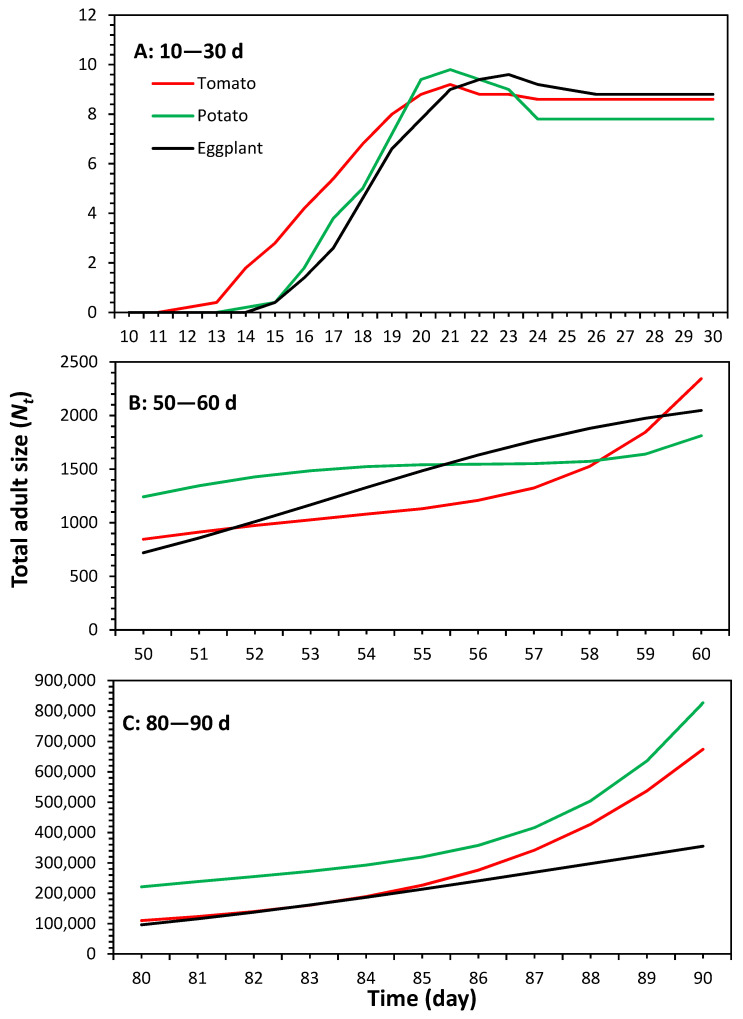
The total adult size (*N_t_*) of *Phenacoccus solenopsis* on different host plants in time intervals (**A**) 10–30 d, (**B**) 50–60 d and (**C**) 80–90 d.

Population growth predictions of *P. solenopsis* on considered host plants generated via the TIMING-MSChart^®^ program are shown in Figure 5, which reveals considerable growth curves. Simulations suggest that they start to appear on the 12th, 14th and 15th days on eggplant, respectively (Figure 6A). The total predicted adult population size (*N_t_*) after 90 days was 674,551 on tomato, 826,717 on potato and 355,139 on eggplant (Figure 6B,C).

## 3. Discussion

Although cotton remains its preferred host, *P. solenopsis* is considered to be a potential economic pest of many other crops [33]. Its host range has been expanding over the last decade alongside its geographic spread largely due to favorable climate change. As a result, many Solanaceous crops including tomato, potato and eggplant, have become common hosts for this species in several newly colonized countries such as Algeria, Egypt, Israel, Iran, Italy and Tunisia, and in these newly invaded countries, where cotton is limited or absent, the damage to Solanaceous crops can be severe due, in particular, to its considerable reproductive capacity: *P. solenopsis* may go through many generations per cropping cycle. Furthermore, modelling studies predict that there will be a rise in the number of generations/year of this species, prompted by global warming [6]. In addition, it has been demonstrated that *P. solenopsis* produces more honeydew on tomato than on cotton [34], causing a greater amount of indirect damage, linked to the development of sooty mould which causes a reduction of photosynthesis and a depreciation of fruit quality. The production of honeydew also prompts a mutualism between ants and *P. solenopsis* that contributes to the rapid infestation by the mealybug, also hampering the possible action of natural enemies. For example, it has been demonstrated how ants successfully facilitated the invasion of another mealybug, *Delottococcus aberiae* De Lotto (Hemiptera: Pseudococcidae), on citrus in Spain [35]. In Mediterranean crops, the situation may be even more complicated due to the absence of coevolved specific natural enemies of this pest and the concomitant pressure posed by other invasive pests such as *Tuta absoluta* (Meyrick, 1917) (Lepidoptera: Gelechiidae) (the tomato leafminer) on tomato and eggplant and *Leptinotarsa decemlineata* (Say, 1824) (Coleoptera: Chrysomelidae) (the Colorado potato beetle) on potato [3,14].

Previous studies have shown that host plants can significantly affect the life history parameters and population dynamics of *P. solenopsis* [36,37,38,39] and have a significant impact on *Aenasius bambawalei* (Girault, 1915) (Hymenoptera: Encyrtidae), an efficient solitary endo-parasitoid of this pest, described from India and possibly accidentally introduced into that country along with its host pest [40].

In our study, tomato, potato and eggplant were all found to be suitable hosts for the pest. The theory suggests that the most appropriate host plant for a polyphagous insect pest should allow shorter APOP and TPOP and higher fecundity, net reproductive rate (*R*_0_) and finite rate of increase (*λ*). Nabil [41] reported very high population densities of *P. solenopsis* in open field crops of eggplant in the Hihhya district, Sharqia Governorate in Egypt, reaching 328.75 individuals/leaf during September 2016. High densities of different instars of the pest reaching 150 individuals per plant were also recorded when reared on tomato in Israel by Spodek et al. [11]. In Egypt, natural infestation of the pest on tomato was also reported in Qalyubia governorate [42].

Significant differences in the selected parameters may be reasonably attributed to specific biochemical and morphological features and are in accordance with what has been reported in previous studies. For example, Shahid et al. [43] tested 25 different host plants for the development of *P. solenopsis*, correlating their morphological traits to the population dynamic of the pest. The authors concluded that eggplant is one of the most favorable host plants for the mealybug. Similarly, Nagrare et al. [44] found that the highest net reproductive rate was on cotton (284 females/female/generation) and the lowest was on tomato.

It is reasonable to hypothesize that the abundant glandular trichomes scattered on tomato leaves and stems play a key role in hampering the development of *P. solenopsis*. Nonetheless, it can conclude its cycle even on this plant, thus becoming another serious threat in the whole Mediterranean area.

The potential damage of *P. solenopsis* to eggplant and tomato can be particularly significant when they are grown in greenhouses, where it takes advantage of optimal climatic conditions and high host density. Indeed, Prasad et al. [45] observed the greatest fecundity and survival of crawlers at 30 °C and 32 °C, respectively, which are both common temperatures of protected crops in Mediterranean countries. This situation may lead to the overuse of chemicals in these crops while increasing plant protection costs and disrupting already implemented control schemes for other relevant pests. Furthermore, in Asia (in particular, Pakistan and India) *P. solenopsis* has already developed resistance to a wide range of insecticides including organophosphates, pyrethroids and neonicotinoids due to the continuous and severe use of these compounds on cotton cultivations [19,20,21]. This has severe consequences on efficient chemical control options of this pest on invaded host crops in the Mediterranean Basin since recent phylogenetic analyses performed in Tunisia and Italy revealed that the populations introduced in both countries most probably derive from Asian stock [16,17]. The data presented in this study may be a starting point for the development of suitable agroecological management strategies, such as those based on inter- and border cropping, with a view to progressive reduction of pest populations on cultivated crops and with the enhancement of biological control. These strategies are mostly needed in relation to reducing the application of synthetic insecticides as required by the European Union and by consumers.

## 4. Materials and Methods

### 4.1. Plants

Plants used in this study were tomato (Variety ‘Dorra’), potato (Variety ‘Spunta’) and eggplant (Variety ‘Tizona’) grown in a glasshouse from seed (or tuber) in plastic trays (330 mm × 250 mm × 130 mm) in peat substrate, watered with tap water every two days and maintained under identical natural conditions without fertilizers and chemicals until they reached 150 mm height.

### 4.2. Insects

A laboratory rearing of *P. solenopsis* was initiated at the High Agronomic Institute of Chott-Mariem, Sousse, Tunisia, using specimens collected on *Lantana camara* L. (Verbenaceae) in Tunis, Tunisia, in spring 2022. The identity of specimens was confirmed using both morphological and molecular approaches [17]. Initially, the mealybug was reared for five generations on potato plants (Variety ‘Spunta’) obtained from tubers placed in plastic containers (330 mm × 250 mm × 130 mm) filled with fine sand. To avoid possible effects of host shifting during the experiments, two other colonies were set up using individuals reared on potato, on tomato and on eggplant. All colonies were maintained for five generations in a climatic chamber at 25 ± 2 °C, 60–70% RH and 16:8 h (L:D) photoperiod before their use in the bioassay.

### 4.3. Experimental Protocol

For each host plant (tomato, potato and eggplant), 50 plants were transplanted individually into plastic cups with 200 mL peat substrate. Peer cohorts of *P. solenopsis* were used to collect eggs and a single freshly laid egg (<1 h) was transferred to the central vein of an apical leaf using a fine soft paintbrush under a binocular microscope (Leica^®^ MZ8, Leica Microsystems, Wetzlar, Germany). Plants bearing eggs were then incubated in a climatic cabinet (Scimmit, Shanghai Scimmit Technology, Shanghai, China) at 25 ± 1 °C temperature, 60 ± 5% relative humidity and 16:8 h (L:D) photoperiod. Plants were watered daily with tap water using a 50 mL volume syringe. The entire life cycle of each mealybug individual on each plant was monitored daily until its death. The moults during the larval stages were recorded by the presence of exuviae. Newly emerged adults were kept as couples to record the following parameters: egg incubation period, duration of each immature stage, pre-oviposition period, oviposition period, fecundity, post-oviposition period, adult sex and adult longevity.

### 4.4. Demographic Analyses

Collected data on the development and reproduction on each considered host plant were analyzed according to the age-stage two-sex life table theory as described by Chi and Liu [27] and Huang and Chi [46]. We calculated age-stage-specific survival rate (*s_xj_*: the probability that a newly laid egg will survive to age *x* and stage *j*), age-stage-specific fecundity (*f_xj_*: the mean fecundity of females at age *x*), age-specific survival rate (*l_x_*: the probability that a newly laid egg will survive to age *x*), and age-specific fecundity (*m_x_*: the mean fecundity of individuals at age *x*) (Supplementary Materials S1).

The means and standard errors of the life table parameters were estimated using the bootstrap method with a bootstrap number of m = 100,000 in order to ensure precise estimates [47]. TWOSEX-MSChart^®^ [48] for Windows^®^ (Version 2023.12.15) was used to construct and analyze age-stage two-sex life tables. A paired bootstrap test within TWOSEX-MSChart^®^ was used to compare differences in developmental time, adult longevity, adult preoviposition period (APOP), total preoviposition period (TPOP), oviposition days and fecundity between treatments. The population parameters were also compared using the paired bootstrap test, based on the confidence interval of difference [47,49]. The *p* values of the paired bootstrap test were defined as follows: *P_TP_*, tomato to potato; *P_TE_*, tomato to eggplant; *P_PE_*, potato to eggplant.

### 4.5. Population Projection

The TIMING-MSChart^®^ [50] program (Version 04/18/2024) was used to simulate population growth rate and the structure of each age-stage of *P. solenopsis* over a period of 90 days with an initial population of 10 eggs and without control.

## Figures and Tables

**Table 1:** Developmental duration and adult longevity of *Phenacoccus solenopsis* on different host plants (25 ± 1 °C, 60 ± 5% RH and 16:8 h (L:D)). Values are means ± standard errors. Means in a row followed by different letters are significantly different at *p* < 0.05 using paired bootstrap test.

Developmental Duration (Days)	Host Plants		
	Tomato	Potato	Eggplant
Egg incubation	1 ± 0.00 a (*n* = 50)	1 ± 0.00 a (*n* = 50)	1 ± 0.00 a (*n* = 50)
First-instar nymph			
Female	4.8837 ± 0.1323 c (*n* = 27)	5.5897 ± 0.1132 b (*n* = 28)	6.1818 ± 0.1216 a (*n* = 23)
Male	4.7142 ± 0.3619 b (*n* = 23)	5.3636 ± 0.1511 b (*n* = 22)	6.50 ± 0.4320 a (*n* = 27)
Second-instar nymph			
Female	4.7441 ± 0.1870 a (*n* = 26)	4.8461 ± 0.1835 a (*n* = 26)	4.7045 ± 0.1564 a (*n* = 23)
Male	5.2857 ± 0.4755 b (*n* = 23)	6.5454 ± 0.4103 a (*n* = 22)	5.1666 ± 0.4819 b (*n* = 27)
Third-instar			
Female nymph	6.3023 ± 0.2688 b (*n* = 26)	6.2820 ± 0.2084 b (*n* = 26)	7.0909± 0.2417 a (*n* = 22)
Male pupa	6.7142 ± 0.4751 a (*n* = 23)	7.0909 ± 0.5436 a (*n* = 22)	4.50 ± 0.2249 b (*n* = 27)
Total pre-adult			
Female	16.9302 ± 0.3591 b (*n* = 26)	17.7179 ± 0.2493 b (*n* = 26)	18.9772 ± 0.2868 a (*n* = 22)
Male	17.7142 ± 0.8108 b (*n* = 23)	20.0000 ± 0.1899 a (*n* = 22)	17.1666 ± 0.6044 b (*n* = 27)
Adult longevity			
Female	21.0697 ± 0.3421 b (*n* = 26)	19.8205 ± 0.1197 c (*n* = 26)	24.1590 ± 0.5029 a (*n* = 22)
Male	3.1428 ± 0.1437 b (*n* = 23)	3.1818 ± 0.2248 b (*n* = 22)	6.5000 ± 0.3457 a (*n* = 27)
Total life cycle			
Female	38.0000 ± 0.4319 b (*n* = 26)	37.5384 ± 0.2466 b (*n* = 26)	43.1363 ± 0.5683 a (*n* = 22)
Male	20.8571 ± 0.8877 b (*n* = 23)	23.1818 ± 0.3234 a (*n* = 22)	23.6666 ± 0.7670 a (*n* = 27)

**Table 2:** Fecundity, adult preoviposition period (APOP), total preoviposition period (TPOP) and oviposition days of *Phenacoccus solenopsis* on different host plants (25 ± 1 °C, 60 ± 5% RH and 16:8 h (L:D)). Values are means ± standard errors. Means in a row followed by different letters are significantly different at *p* < 0.05 using paired bootstrap test.

Parameters	Host Plants		
	Tomato	Potato	Eggplant
APOP (days)	6.3953 ± 0.2162 b	5.2051± 0.1964 c	10.0909 ± 0.5255 a
TPOP (days)	23.3256 ± 0.405 b	22.9222 ± 0.3091 b	29.0682 ± 0.5807 a
Fecundity (eggs)	155.6046 ± 11.9904 c	244.9230 ± 9.7554 b	276.5000 ± 10.7814 a
Oviposition (days)	12.7209 ± 0.2469 b	13.6410 ± 0.1725 a	10.8409 ± 0.1109 c

**Table 3:** Net reproductive rate (*R*_0_), the intrinsic rate of increase (*r*), finite rate of increase (*λ*) and generation time (*T*) of *Phenacoccus solenopsis* on different host plants (25 ± 1 °C, 60 ± 5% RH and 16:8 h (L:D)). Values are means ± standard errors. Means in a row followed by different letters are significantly different at *p* < 0.05 using the paired bootstrap test.

Parameters	Host Plants		
	Tomato	Potato	Eggplant
*R*_0_	133.82 ± 12.7829 c	191.04 ± 16.23395 b	243.32 ± 15.83068 a
*r*	0.1731 ± 0.00424 b	0.1860 ± 0.0039 a	0.1696 ± 0.00364 b
*λ*	1.1889 ± 0.005042 a	1.2044 ± 0.00469 b	1.1848 ± 0.00431 a
*T*	28.2854 ± 0.4532 b	28.2371 ± 0.3660 b	32.3899 ± 0.5958 a

## Data Availability

The datasets used and analyzed during the current study are available from the corresponding author upon reasonable request.

## Supplementary Materials

The following supporting information can be downloaded at: https://www.mdpi.com/article/10.3390/plants13101381/s1. References [51,52,53,54,55] are cited in the supplementary materials.
