# Supplementary material for: Using Age-Stage Two-Sex Life Tables to Assess the Suitability of Three Solanaceous Host Plants for the Invasive Cotton Mealybug Phenacoccus solenopsis Tinsley
Source: Plants (Basel). 2024 May 16;13(10):1381. doi: 10.3390/plants13101381 (PMC11125115; doi:10.3390/plants13101381)
Supplement: Supplementary file 1 [file plants-13-01381-s001.zip › plants-2973755-supplementary.pdf]

plants

MDPI

Supplementary Materials

Using age-stage two-sex life tables to assess the suitability of three Solanaceous host plants for the invasive cotton mealybug *Phenacoccus solenopsis* Tinsley

Khaled Abbes, Ahlem Harbi, Emilio Guerrieri and Brahim Chermiti

We calculated age-stage specific survival rate (s_{xj}: the probability that a newly laid egg will survive to age x and stage j), age-stage specific fecundity (f_{xj}: the mean fecundity of females at age x), age-specific survival rate (l_x: the probability that a newly laid egg will survive to age x), and age-specific fecundity (m_x: the mean fecundity of individuals at age x). In the age-stage two-sex life table, l_x and m_x are calculated as [27-28]:

$$l_x = \sum_{j=1}^{k} s_{xj}$$

(S1)

$$m_x = \frac{\sum_{j=1}^{k} s_{xj} f_{xj}}{\sum_{j=1}^{k} s_{xj}}$$

(S2)

where k is the last stage of the study cohort. The net reproductive rate (R_0) represents the total number of offspring that an individual can produce during its lifetime and is calculated as [27-28]:

$$R_0 = \sum_{x=0}^{\infty} l_x m_x$$

(S3)

The intrinsic rate of increase (r) is estimated by using the iterative bisection method from the Euler-Lotka equation with age indexed from zero as follows [27-28]:

$$r = \sum_{x=0}^{\infty} e^{-r(x+1)} l_x m_x = 1$$

(S4)

The finite rate of increase (λ) is calculated as follows [51]:

$$\lambda = e^r$$

(S5)

The mean generation time (T) is defined as the time that a population requires to increase to the R0-fold of its size at the stable age-stage distribution, and is calculated as follows [51]:

$$T = \frac{\ln R_0}{r}$$

(S6)

Age-stage life expectancy (e_{xj}) is the time that an individual of age x and stage j is expected to live and was calculated according to the method described by [52] as:

$$e_{xj} = \sum_{i=x}^{\infty} \sum_{j=y}^{k} s'_{iy}$$

(S7)

where n is the number of age groups, m is the number of stages, and s'_{ij} is the probability that an individual of age x and stage j will survive to age i and stage y.

The age-stage reproductive value (v_{xj}) is defined as the contribution of individuals of age x and stage j to the future population. In the age-stage two-sex life table, it is calculated as [53-55]:

$$V_{xj} = \frac{e^{r(x+1)}}{s_{xj}} \sum_{i=x}^{\infty} e^{-r(i+1)} \sum_{j=y}^{k} s'_{iy} f_{iy}$$

(S8)
